# Five great food clusters of specific IgG for 44 common food antigens. A new approach to the epidemiology of food allergy

**DOI:** 10.1186/2045-7022-3-S3-P67

**Published:** 2013-07-25

**Authors:** AF Speciani, J Soriano, MC Speciani, G Piuri

**Affiliations:** 1 Food Allergy Department, GEK srl, Milan, Italy; 2 Department of Statistical Science, Duke University, Durham, NC, USA; 3 SMA srl, Milan, Italy

## Background

Studies with mouse models demonstrate 2 pathways of systemic anaphylaxis: a classic pathway mediated by IgE, FcεRI, mast cells, histamine, and platelet-activating factor (PAF) and an alternative pathway mediated by total IgG, FcγRIII, macrophages, and PAF. The former requiring fewer antigens and antibodies than the latter. The importance of the alternative pathway in humans is uncertain, but human IgG, IgG receptors, macrophages, mediators, and their receptors have appropriate properties to support this pathway if enough IgG and antigens are present [1].

## Methods

Specific IgG antibodies for 44 common food antigens in serum were measured in 5010 Italian patients (3795 females and 1215 males; age 44.67± 13.94 years). We use an unsupervised linkage procedure to identify clusters among the 44 products. This agglomerative hierarchical algorithm intially has as many groups as variables, and then groups are gradually fused together in function of their similarities. The algorithm stops when all products belong to a single cluster. We use the sample covariance as similarity matrix between the variables, and a complete linkage method to measure distances between groups. This notion of distance guarantees that all the products in a cluster are within a maximum distance of each other.

## Results

We identified five great food clusters. The first group contains dairy products starting from goat and cow milk as well as Parmesan cheese, mozzarella and ricotta cheese. The second group consists in foods with higher concentration of nickel salts such as tomatoes, spinach, oat, buckwheat and so on. Within this group it is possible to identify the third cluster about wheat and related grains such as kamut. The fourth group is about yeasts such as Aspergillus fumigatus and Saccharomyces cerevisiae and contains also porcini mushrooms and champignon mushrooms. This cluster is probably connected with fermented foods. In the last group appear foods with the higher concentration of natural salicylates: products such as honey, tea, courgette, orange and so on.

## Conclusion

The identification of these five great food clusters is probably connected with different diet habits. Once the importance of the anaphylaxis alternative pathway in humans subjects is clarified, this knowledge will be useful in diagnosis and therapy of non-IgE-mediated allergic forms.

## Disclosure of interest

None declared.
